# Semi-Automated Detection of Cerebral Microbleeds on 3.0 T MR Images

**DOI:** 10.1371/journal.pone.0066610

**Published:** 2013-06-21

**Authors:** Hugo J. Kuijf, Manon Brundel, Jeroen de Bresser, Susanne J. van Veluw, Sophie M. Heringa, Max A. Viergever, Geert Jan Biessels, Koen L. Vincken

**Affiliations:** 1 Image Sciences Institute, University Medical Center Utrecht, Utrecht, The Netherlands; 2 Department of Neurology, Rudolf Magnus Institute of Neuroscience, University Medical Center Utrecht, Utrecht, The Netherlands; 3 Department of Radiology, University Medical Center Utrecht, Utrecht, The Netherlands; Cornell University, United States of America

## Abstract

Cerebral microbleeds are associated with vascular disease and dementia. They can be detected on MRI and receive increasing attention. Visual rating is the current standard for microbleed detection, but is rater dependent, has limited reproducibility, modest sensitivity, and can be time-consuming. The goal of the current study is to present a tool for semi-automated detection of microbleeds that can assist human raters in the rating procedure. The radial symmetry transform is originally a technique to highlight circular-shaped objects in two-dimensional images. In the current study, the three-dimensional radial symmetry transform was adapted to detect spherical microbleeds in a series of 72 patients from our hospital, for whom a ground truth visual rating was made by four raters. Potential microbleeds were automatically identified on T2*-weighted 3.0 T MRI scans and the results were visually checked to identify microbleeds. Final ratings of the radial symmetry transform were compared to human ratings. After implementing and optimizing the radial symmetry transform, the method achieved a high sensitivity, while maintaining a modest number of false positives. Depending on the settings, sensitivities ranged from 65%–84% compared to the ground truth rating. Rating of the processed images required 1–2 minutes per participant, in which 20–96 false positive locations per participant were censored. Sensitivities of individual raters ranged from 39%–86% compared to the ground truth and required 5–10 minutes per participant per rater. The sensitivities that were achieved by the radial symmetry transform are similar to those of individual experienced human raters, demonstrating its feasibility and usefulness for semi-automated microbleed detection.

## Introduction

Cerebral microbleeds are small spherical lesions that are visible as hypointensities on T2*-weighted MR images. Recent studies showed that microbleeds are associated with cerebrovascular disease and dementia. [1] Therefore, interest in microbleeds is increasing rapidly and they are being investigated in large studies (e.g. the population-based Rotterdam Scan Study [2]). The current standard for detection of microbleeds on T2*-weighted MR scans is rating with validated visual rating scales, for example the Microbleed Anatomical Rating Scale (MARS) or the Brain Observer MicroBleed Scale (BOMBS). [3], [4] However, this process is rater dependent, has limited reproducibility, a modest sensitivity, and can be time-consuming. [4], [5].

Semi-automated techniques for the detection of cerebral microbleeds are likely to improve the aforementioned issues. These techniques can reduce rater dependence and improve sensitivity, both for experienced and less experienced raters. Other potential advantages are a reduction in required rating time and a higher reproducibility. As such, semi-automated detection may be especially useful for large research studies, in which higher field strengths, higher spatial resolution of scans, and a higher required sensitivity increase the rating difficulty and rating time [5].

Several methods currently exist for the semi-automated detection of microbleeds. Seghier *et al.* [6] described a method that uses a unified segmentation-normalization approach to detect microbleeds. This method detected 50% of the microbleeds present in their participants. While the number of false positives was not reported, manual removal of the false positives required 5–10 minutes on average per participant. Barnes *et al.* [7] used a combination of statistical thresholding and a support vector machine supervised learning classifier on susceptibility weighted images. This method had a sensitivity of 82%. On average, over 100 false positives were found per participant, which takes a human rater 5–15 minutes to censor. In a previous study [8], we used the 3D radial symmetry transform (RST) to detect microbleeds on non-clinical dual-echo 7.0 T gradient echo data. The 3D RST had a sensitivity of 71%, which is higher than the sensitivity of individual human raters on 7.0 T scans. On average, 17 false positives were found per participant, requiring 2 minutes to remove, as opposed to 30 minutes for a full manual rating.

Since clinical use of ultra-high field strength (7.0 T) MRI is still limited, the ability to apply the RST on 3.0 T MR images would be highly useful. Such images are more widely available, but this comes with some challenges, including multi-slice scanning protocols, lower spatial resolution, and an anisotropic voxel size.

In the present study, the use of the RST on 3.0 T T2*-weighted images was investigated. The approach of the RST, detecting hypointense spherical objects using uncomplicated image processing techniques, combined with its excellent results on dual echo 7.0 T scans forms an ideal starting base for the detection of microbleeds on 3.0 T images. In order to successfully apply the RST on 3.0 T images, the aforementioned challenges and the lack of additional echoes had to be overcome. The goal was to develop a practical tool for semi-automated detection of microbleeds that can support human raters, offering a high sensitivity while minimizing the number of false positives that have to be censored.

## Materials and Methods

### Participants

For the present study, 72 patients (mean age: 77 years, sd: 8 years, 38 men, 34 women) were included from a consecutive series of patients referred to the memory clinic of the University Medical Center Utrecht, the Netherlands. Inclusion criteria for this study cohort were: cognitive complaints, Mini-Mental State Examination [9] score of 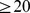 and Clinical Dementia Rating ≤1. [10] Exclusion criteria were: contraindications for MRI (e.g. pacemaker, claustrophobia), a psychiatric or neurological disorder that could influence cognitive functioning, recent non-disabling stroke (>2 years) or any disabling stroke, major depression or a history of alcohol or substance abuse. All 72 patients underwent an MRI-examination between November 2009 and June 2011. Although some scans contained motion artifacts, no patients were excluded for that reason. Diagnoses were established at a multidisciplinary meeting, including: subjective cognitive complaints (n = 9), mild cognitive impairment (according to Petersen criteria [11], n = 23), possible (n = 4) and probable (n = 33) Alzheimer's Disease (according to the clinical criteria of the National Institute of Neurological Disorders and Stroke-Alzheimer's Disease and Related Disorders Association [12]), vascular dementia [13] (n = 1) and semantic dementia [14] (n = 2).

The study was approved by the medical ethics committee of the University Medical Center Utrecht. Written informed consent was given by all participants.

### MRI Acquisition

The participants underwent a standardized MR exam on a 3.0 T Philips Achieva MR scanner using an eight-channel head coil, including, among others, a multi-slice T2*-weighted sequence (TR: 1653 ms, TE: 20 ms), a multi-slice FLAIR sequence (TR: 11000 ms, TE: 125 ms, TI: 2800 ms), and a 3D T1-weighted turbo field echo sequence (TR: 7.9 ms, TE: 4.5 ms). The T2*-weighted and FLAIR sequences were reconstructed to a voxel size of 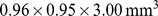. The T1-weighted sequence was reconstructed to a voxel size of 1.0 mm isotropic.

### Study Design

In the current study, we used the RST that was originally designed for 7.0 T dual-echo gradient echo images and had a high accuracy. [8] Because there are still relatively few ultra-high field strength MR scanners available around the world, the ability to apply this method on (single echo) 3.0 T T2*-weighted images leads to higher generalization and opportunities for its use.

First, microbleeds were visually rated on the scans of all participants by four human raters. Based on these ratings, a visual ground truth was created. Second, the RST was implemented for the use on 3.0 T MR scans. Five participants with microbleeds were randomly selected and used to determine suitable parameters for the RST and to optimize the work flow. Next, the output of the RST had to be thresholded, to distinguish potential microbleeds from false positives. A series of thresholds was applied and the results were evaluated using a free-response receiver operating characteristic (FROC) curve. Finally, a human rater used a user-friendly interface to inspect the results of the RST after thresholding and censored any remaining false positives. If extra positives were detected by the RST (i.e. microbleeds that were not in the original ground truth), these were added to form the final ground truth if the majority of raters confirmed them as true microbleeds.

### Visual Rating of Microbleeds

Microbleeds were rated visually on the T2*-weighted scans of all participants by four raters with different degrees of experience, blinded to all other clinical information. All raters were sufficiently trained prior to the rating sessions.

Rating was performed according to the MARS, as described by Gregoire *et al.* [3] In this rating scale, ‘definite’ microbleeds are defined as hypointense round lesions. If a rater was uncertain about a lesion being a microbleed, it was rated as ‘possible’. Mimics like symmetric calcifications in the basal ganglia were disregarded. The detected microbleed locations were compared between raters and in case of discordance, a majority decision was made or an experienced fifth rater was consulted. This consensus-based lesion rating formed the visual ground truth rating.

### Radial Symmetry Transform

The RST is an image processing technique that can be used to highlight spherical-shaped objects in an image. It has numerous applications, both medical and non-medical. For example, the detection of nuclei in H&E stained breast cancer biopsy images and the detection of eyes in pictures of human faces. [15], [16] Recently, a 3D version of the RST was used for automatic detection of microbleeds on 7.0 T MR images. [8].

Before applying the RST, a binary mask of the gray and white matter was obtained using unified segmentation as implemented in SPM8. [17] For this, the T1-weighted sequence was used and the resulting mask was transformed to the T2*-weighted sequence using elastix. [18] Within this mask, the intensities of the T2*-weighted sequence were normalized using a histogram range-matching procedure. [19] Inspection of the intensities of microbleeds present in the selected five participants showed that all intensity values were below the 6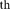 percentile of the histogram. This was used to normalize the intensity values to a range of [0, 255] using the 6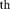 and 95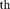 percentile.

**Figure 1:**
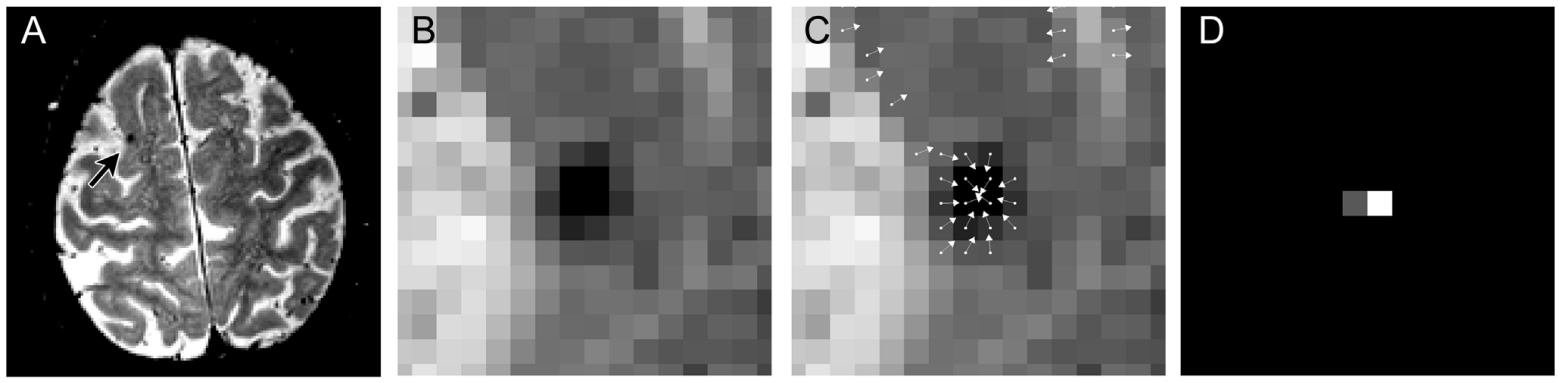
Workflow of the radial symmetry transform. An example slice of a 3.0 T T2*-weighted image with a microbleed is shown in A. B shows a zoom-in of the microbleed shown in A. An intermediate step of the radial symmetry transform is shown in C, where each voxel contributes to some neighboring voxel. If multiple arrows point towards the same target voxel, this target voxel receives a high radial symmetry value. The output of the radial symmetry transform is shown in D, displaying a large value at the center voxel of the microbleed.

For each voxel in the image, the 3D RST resulted in a so-called radial symmetry value. This value corresponds to the sphericalness of a local neighborhood around the voxel. In the case of a microbleed, the center voxel of the microbleed will receive a high radial symmetry value, because the surrounding hypointense voxels form a spherical spot. This is illustrated in Figure 1.

After computation of the 3D RST, a threshold value 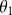 was set on the radial symmetry value. Locations within the mask with a value above 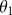 were considered potential microbleed locations, thereby removing locations that have no radial symmetry (e.g. normal brain tissue) or low radial symmetry (e.g. vessels). This procedure is similar to the method described earlier for detection of microbleeds on 7.0 T dual-echo gradient echo images [8]. However, the lower spatial resolution of the 3.0 T images and the lack of a second echo posed a challenge: the number of false positives remaining after the previous steps was significantly higher (up to 200 per participant). The availability of the second echo at 7.0 T enabled the removal of false positive locations that were only visible on one echo, but this was not possible for the 3.0 T images. Therefore, an additional step to the method was introduced for 3.0 T images after computation of the 3D RST, involving a 2D RST computed on a transversal minimum intensity projection (minIP) image. The rationale behind this additional step was derived from the visual rating process of microbleeds, in which a rater searches for 2D hypointense circular spots on a minIP representation of the scan. On a minIP, microbleeds show up as dark round spots, while typical false positives (such as vessels) are displayed as elongated structures. This facilitates the distinction between microbleeds and vessels.

A minIP slab of 12 mm thickness was created in a square region of interest (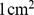) at each potential microbleed location after the 3D RST. The 2D RST was computed on this transversal slab with settings identical to the 3D RST. If the new radial symmetry value of the potential microbleed did not exceed the threshold 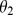, it was discarded as a potential microbleed.

### Experiments

Parameter settings that influence the quality of the 3D RST were optimized on the selected five participants with microbleeds, similar to a study published earlier. [20].

Threshold values 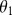 and 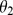 were used after computation of the 3D and 2D RST, respectively. These threshold values determined the locations that were considered as potential microbleeds. Different values gave different results and therefore a range of threshold values was heuristically established. This was done on the selected five participants with microbleeds. Subsequently, an exhaustive search that computed the results of the RST for all combinations of 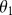 and 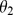 was performed on all 72 participants.By plotting a FROC curve with the results of this search, the sensitivity of each combination of 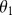 and 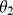 can be assessed together with the number of false positives.

Three combinations of 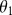 and 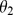 were selected, representing: *A*) moderate, *B*) good, and *C*) high sensitivity for detection of microbleeds. For the three combinations, rater 4 censored all potential microbleed locations that were identified. The censoring of the potential microbleed locations was performed several months after the initial visual rating of rater 4, to ensure that previous ratings were not remembered. Additionally, if microbleeds were detected by the RST that were not present in the original visual rating, they were presented to all human raters for inspection. If at least three raters had confirmed them as true microbleeds, they were added as ‘extra positives’ to the final ground truth rating. Adding the extra microbleeds improved the ground truth rating and gave a fair comparison of the sensitivities of human raters versus the RST.

## Results

During the visual rating, 244 locations were marked as definite or possible microbleeds by at least one of the four raters. After the consensus meeting, 148 locations in 38 participants (53%) were confirmed as microbleeds and defined as the visual ground truth: 106 definite and 42 possible microbleeds. Of those microbleeds: 93 (63%) were detected by rater 1, 130 (88%) by rater 2, 60 (41%) by rater 3, and 104 (70%) by rater 4.

To determine the appropriate range of 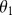 and 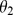, the radial symmetry values at the locations of microbleeds in the five participants were inspected. As a result, 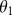 was chosen to range from 0.5 to 3.0 and 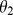 from 0.0 to 3.0, both with a step size of 0.5. Setting 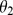 to zero means that the 2D RST was not computed at all.

**Figure 2:**
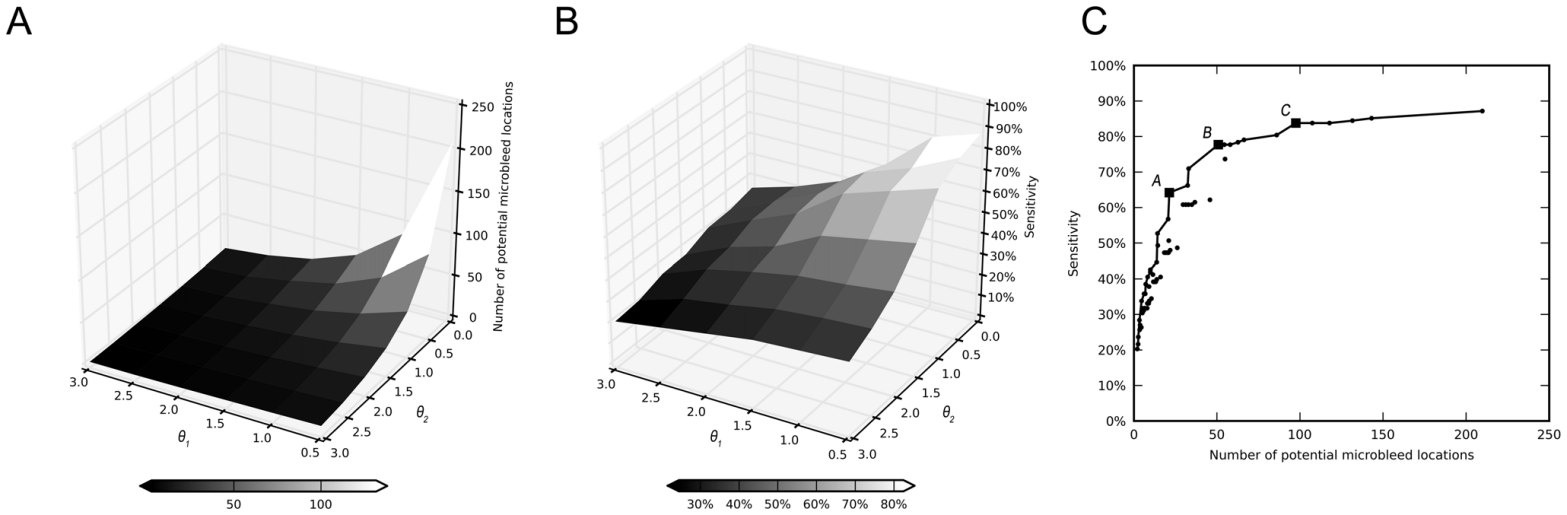
Results of the radial symmetry transform for detecting microbleeds. Appropriate values for 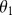 and 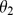 were determined on five randomly selected participants with microbleeds. The figures show the results of these values on all 72 participants. A) Number of detected potential microbleed locations per participant (n = 72), visualized as a function of 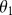 and 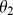. Potential microbleeds need to be censored by a human rater to identify true microbleeds (on average 2 per participant) and reject false positives that remained after the 3D and 2D radial symmetry transform. B) Sensitivity of the radial symmetry transform on the visual ground truth, visualized as a function of 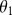 and 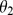. C) This figure shows the relationship between sensitivity and the number of potential microbleed locations per participant, where each dot is a combinations of 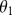 and 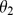. Depending on the preferred sensitivity, there is an optimal combination of 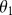 and 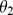 with the lowest number of locations. These optimal combinations are annotated by the solid line. Three combinations (*A*, *B*, and *C*, annotated from left to right with squares), with moderate, good, and high sensitivity, were selected for inspection by a human rater.

For each combination of 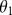 and 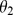, the number of potential microbleed locations per participant is shown in Figure 2A and the sensitivity of the method is shown in Figure 2B. These figures clearly show that lower values of 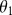 and 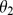 result in more potential microbleed locations (i.e. a higher false positive rate), but also a higher sensitivity. Therefore, when searching for the optimal 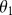 and 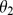, a trade-off has to be made between a high sensitivity and a long censoring time, or a lower sensitivity with a shorter censoring time. This is illustrated in Figure 2C, where all 42 combinations of 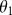 and 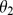 are plotted in a FROC curve. As the sensitivity increases, the number of false positives increases accordingly. The solid line in the graph indicates the optimal combinations of 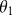 and 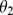, i.e. the highest sensitivity having the lowest number of false positives.

Based on this graph, three combinations of 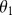 and 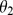 (*A*, *B*, and *C*) were selected and presented to rater 4 for censoring. The results of the semi-automatic method after manual censoring are shown in Table 1.

**Table 1:** Results of the radial symmetry transform after censoring potential microbleed locations per participant.

	Number of microbleeds				Required rating time (minutes)			
	#TP	#EP	#FP	(mean±sd)	Total	Median	Q1	Q3
A	95	4	1436	(20±12)	70.8	0.7	0.4	1.1
B	115	4	3538	(49±25)	121.0	1.4	0.9	1.9
C	124	4	6891	(96±44)	197.5	2.2	1.9	3.3

Total number of detected true positives, extra positives, false positives, and total time required to censor the false positives in all participants. Median rating time required by rater 4, with the interquartile range (Q1 and Q3), is shown. In the visual ground truth rating, 148 microbleeds were found in the 72 participants.

In total, 4 extra positives (all possible microbleeds) were found that were not present in the visual consensus rating, but were marked as potential microbleeds by the RST and thereafter confirmed by the majority of raters. These were added to the final ground truth, resulting in a total of 152 microbleeds. The results are shown in Table 2.

**Table 2:** Sensitivity of manual and semi-automated rating.

	Sensitivity (count)
Rater 1	61% (93)
Rater 2	86% (130)
Rater 3	39% (60)
Rater 4	68% (104)
All raters	97% (148)
*RST A*	65% (99)
*RST B*	78% (119)
*RST C*	84% (128)

Final sensitivities of the individual human raters, all raters combined, and the three chosen combinations of the radial symmetry transform. A total of 152 microbleeds was found (148 during visual rating +4 extra positives by the radial symmetry transform) in 38 participants (53%).

Of the 38 participants that had at least one microbleed, 30 were identified at setting *A* (i.e. an identification rate of 79%). All eight participants that were not identified had only one microbleed. If a participant had at least one true microbleed detected at setting *A* and subsequently setting *C* was used on only these participants, the final sensitivity of the method for the detection of individual microbleeds was 83%. This enables fast rating, as participants without microbleeds can be quickly discarded at setting *A*, while still achieving a high sensitivity by subsequently using setting *C*.

The time needed for the original visual rating was approximately 5 to 10 minutes per participant per rater. The required human rater time for visual censoring of the results of the RST is reported in Table 1, medians ranged from 1 to 2 minutes per participant. A dedicated user-friendly interface supported the human rater to censor the results of the RST. Time required for loading the scan into the computer memory, adjusting the contrast settings of the scan prior to rating to normalize the viewing, and breaks were not taken into account. Computation time of the 3D RST was approximately 20 seconds per participant. The computation time of the 2D RST depended on the number of potential microbleeds, influenced by the values of 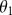 and 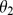, and required another 5 seconds to 2 minutes per participant. All computations were performed on a standard workstation.

## Discussion

Detection of microbleeds on T2*-weighted 3.0 T MRI scans can be achieved by the RST, with a high sensitivity and a limited amount of visual rating time required to censor false positive locations.

Most importantly, the sensitivity of the method was similar to the sensitivity of the most experienced human raters. This would allow for the method to be used in practice, for example in research studies to assess the presence of microbleed in large patient cohorts. The use of the method will make the detection of microbleeds less rater dependent and thereby improve the overall quality of the rating.

Comparing the presented method and its results to existing methods, the RST shows some advantages. The resulting sensitivities of 65%–84% are higher than the reported sensitivities of the methods of Seghier *et al.* (50%) and Barnes *et al.* (82%). It should be noted that all methods did not include a full FROC curve in their results. Therefore it could be that other sensitivities might be possible and that the presented results are only a single point on the FROC curve, being a tradeoff between sensitivity and number of false positives. Furthermore, other factors, such as differences in scan type and quality, might explain differences between various methods.

Recently, Bian *et al.* demonstrated the use of the 2D RST for the detection of radiation-induced microbleeds on 3.0 T susceptibility-weighted images. [21] The images underwent a minIP post-processing on which the 2D RST was applied and additional image processing techniques were used to eliminate false positive locations. This resulted in a sensitivity of 87% with on average 45 false positive locations per participant. The work of Bian and the method presented here demonstrate the effectiveness of the RST for microbleed detection. Although there are some differences in the approach, participant population, and cause of microbleeds, these works contribute to the understanding of applying the RST for microbleed detection and complement each other.

Comparing the required human rater time showed lower rating times with the RST, requiring only 1–2 minutes as compared to 5–10 min. (Segier *et al.*) and 5–15 min. (Barnes *et al.*) Bian *et al.* did not report the required rating time, but only the average numbers of 30 true microbleeds and 45 false positive locations per participant. Seghier *et al.* did not report the number of detected potential microbleed locations, so the rating time cannot be explained. Barnes *et al.* reported on average 125 potential microbleed locations per participant, with on average 17 true positives and 108 false positives. The longer rating time as compared to the RST might be caused by the higher number of true microbleeds that were present. During the censoring of the results of the RST, rater 4 needed more time to correctly identify a true microbleed location than to reject a false positive location. Assuming this behavior also holds for the raters in the study of Barnes *et al.*, this might explain the longer required human rater time. Next to this, false positives of the RST appeared mostly at locations where they could be easily rejected by a trained human rater. Furthermore, individual differences between human raters can also have an effect on the required rating time. Besides this, it is noticeable that censoring the results of the RST on 3.0 T scans required less time than censoring the results of the RST on 7.0 T scans. [8] This is likely caused by the availability of the second echo on 7.0 T scans, that required additional time for inspection. In addition, microbleeds appear smaller on 7.0 T scans and thus require additional zooming by the human rater during censoring.

The most significant advantage of the RST over existing methods is the easy and uncomplicated implementation. The main unique features of a microbleed (circular shape and hypointense on a T2*-weighted scan) are directly translated into the RST. Loy and Zelinsky provide a clear and straightforward description of the implementation of the RST and offer open source implementations online. [16] Furthermore, the method is (largely) independent of scan parameters and field strength of the MR, thus making it generic and suitable for widespread usage. The required amount of training data is minimal, whereas techniques using unified segmentation or classifiers require a substantial amount of training data. Training and optimization of parameters was performed on just a few participants, as demonstrated here and earlier. [20] After the initial training and optimization of the parameters, the entire procedure can be automated and processed offline: extracting images from the picture archiving and communication system, computing the 3D RST, and computing the 2D RST. After these automated steps, limited human rater time is required only for visual censoring of the results using a user-friendly interface.

Since the automatic part of the method always produces the same results, the reproducibility of the method is high but still dependent on the rater that performs the final visual censoring. However, the rater dependence that is usually involved with visual rating of microbleeds can be reduced to a large extent. Potential microbleed locations are annotated by the RST, but identifying true microbleeds and rejecting false positives remains up to the decision and interpretation of a human rater. Less experienced raters now have a tool that will guide them to achieve high sensitivities and experienced raters will benefit from the reduced rating time.

Some microbleeds that were detected by the original visual rating were missed by the RST, even at the setting with a relatively high number of false positives (setting *C*). Most of those false negatives were too small in size to be detected by the RST.

The false positive locations that were detected by the RST needed to be removed by a human rater. These false positive locations were mostly vessels (e.g. periventricular, in sulci, in the cerebellum); calcifications in the basal ganglia and choroid plexus; the interhemispheric fissure and Sylvian fissures; and bone and air artifacts, especially located at the skull base. Scans with motion artifacts had more false positives, but true microbleeds were still reliably detected. Most false positives appeared at locations where they can be easily rejected by a human rater, resulting in a low rating time. Applying the 2D RST removed 50% to 90% of the false positives that were present after the 3D RST, depending on the value of 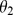. Higher values of 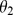 removed more false positives, but introduced some false negatives as well, and vice versa for lower values of 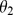 (see Figures 2A and 2B). To remove even more false positives, additional scans such as MR angiography could be included to eliminate vessels.

In our opinion, the ability to adapt and fine-tune the RST gives advanced opportunities for its use in a clinical setting. By simply adapting the parameters 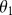 and 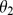, a consideration between sensitivity and rating time can be made. This flexibility of the method can be used to create a time-efficient workflow for microbleed detection in a research setting. With the use of setting *A*, participants without microbleeds can be quickly discarded. Participants with at least one microbleed at setting *A* can be further inspected by using setting *B* or *C*. As such, quick scoring is performed for participants without microbleeds. But for participants with at least one microbleed at setting *A*, a high sensitivity is achieved by using (the more time-consuming) setting *B* or *C*. Another interesting approach would be to apply the RST on quantitative susceptibility mapping (QSM) images. [22] By acquiring multiple echo times, QSM images will be able to provide a better contrast between microbleeds and surrounding tissue. This was not done in the clinical acquisition protocol used, but would probably lead to a higher sensitivity and a lower number of false positives.

### Conclusions

A flexible system for efficient detection of cerebral microbleeds using the radial symmetry transform was presented. The sensitivity of this method is high, while the number of false positives that need to be censored was minimized.

## References

[1] Charidimou A, Werring DJ (2011) Cerebral microbleeds: detection, mechanisms and clinical challenges. Future Neurology 6: 587–611.

[2] Vernooij MW, van der Lugt A, Ikram MA, Wielopolski PA, Niessen WJ, et al (2008) Prevalence and risk factors of cerebral microbleeds: The rotterdam scan study. Neurology 70: 1208–1214. 18378884 10.1212/01.wnl.0000307750.41970.d9

[3] Gregoire SM, Chaudhary UJ, Brown MM, Yousry TA, Kallis C, et al (2009) The microbleed anatomical rating scale (mars): Reliability of a tool to map brain microbleeds. Neurology 73: 1759–1766. 19933977 10.1212/WNL.0b013e3181c34a7d

[4] Cordonnier C, Potter GM, Jackson CA, Doubal F, Keir S, et al (2009) Improving interrater agreement about brain microbleeds: development of the brain observer microbleed scale (bombs). Stroke 40: 94–99. 19008468 10.1161/STROKEAHA.108.526996

[5] de Bresser J, Brundel M, Conijn MM, van Dillen JJ, Geerlings MI, et al.. (2012) Visual cerebral microbleed detection on 7t mr imaging: Reliability and effects of image processing. American Journal of Neuroradiology in press. 10.3174/ajnr.A2960 PMC7964590 22345502

[6] Seghier ML, Kolanko MA, Leff AP, Jger HR, Gregoire SM, et al (2011) Microbleed detection using automated segmentation (midas): A new method applicable to standard clinical mr images. PLoS ONE 6: e17547. 21448456 10.1371/journal.pone.0017547 PMC3063172

[7] Barnes SR, Haacke EM, Ayaz M, Boikov AS, Kirsch W, et al (2011) Semiautomated detection of cerebral microbleeds in magnetic resonance images. Magnetic Resonance Imaging 29: 844–852. 21571479 10.1016/j.mri.2011.02.028 PMC3118856

[8] Kuijf H, de Bresser J, Geerlings M, Conijn M, Viergever M, et al (2012) Efficient detection of cerebral microbleeds on 7.0t mr images using the radial symmetry transform. NeuroImage 59: 2266–2273. 21985903 10.1016/j.neuroimage.2011.09.061

[9] Folstein M, Folstein S, McHugh P (1975) "mini-mental state": A practical method for grading the cognitive state of patients for the clinician. Journal of Psychiatric Research 12: 189–198. 1202204 10.1016/0022-3956(75)90026-6

[10] Hughes CP, Berg L, Danziger WL, Coben LA, Martin RL (1982) A new clinical scale for the staging of dementia. The British Journal of Psychiatry 140: 566–72. 7104545 10.1192/bjp.140.6.566

[11] Petersen RC, Smith GE, Waring SC, Ivnik RJ, Tangalos EG, et al (1999) Mild cognitive impairment: Clinical characterization and outcome. Arch Neurol 56: 303–308. 10190820 10.1001/archneur.56.3.303

[12] McKhann G, Drachman D, Folstein M, Katzman R, Price D, et al (1984) Clinical diagnosis of alzheimer's disease. Neurology 34: 939. 6610841 10.1212/wnl.34.7.939

[13] Román GC, Tatemichi TK, Erkinjuntti T, Cummings JL, Masdeu JC, et al (1993) Vascular dementia. Neurology 43: 250. 8094895 10.1212/wnl.43.2.250

[14] Neary D, Snowden JS, Gustafson L, Passant U, Stuss D, et al (1998) Frontotemporal lobar degeneration. Neurology 51: 1546–1554. 9855500 10.1212/wnl.51.6.1546

[15] Veta M, Huisman A, Viergever M, van Diest P, Pluim J (2011) Marker-controlled watershed segmentation of nuclei in h&e stained breast cancer biopsy images. In: Biomedical Imaging: From Nano to Macro, 2011 IEEE International Symposium on. 618–621. doi: 10.1109/ISBI.2011.5872483

[16] Loy G, Zelinsky A (2003) Fast radial symmetry for detecting points of interest. IEEE Transactions on Pattern Analysis and Machine Intelligence 25: 959–973.

[17] Ashburner J, Friston K (2005) Unified segmentation. NeuroImage 26: 839–851. 15955494 10.1016/j.neuroimage.2005.02.018

[18] Klein S, Staring M, Murphy K, Viergever M, Pluim J (2010) elastix: A toolbox for intensity-based medical image registration. Medical Imaging, IEEE Transactions on 29: 196–205. 10.1109/TMI.2009.2035616 19923044

[19] Cocosco CA, Zijdenbos AP, Evans AC (2003) A fully automatic and robust brain mri tissue classification method. Medical Image Analysis 7: 513–527. 14561555 10.1016/s1361-8415(03)00037-9

[20] Kuijf H, de Bresser J, Biessels G, Viergever M, Vincken K (2011) Detecting cerebral microbleeds in 7.0 t mr images using the radial symmetry transform. In: Biomedical Imaging: From Nano to Macro, 2011 IEEE International Symposium on. 758–761. doi:10.1109/ISBI.2011.5872516

[21] Bian W, Hess CP, Chang SM, Nelson SJ, Lupo JM (2013) Computer-aided detection of radiation induced cerebral microbleeds on susceptibility-weighted mr images. NeuroImage: Clinical 2: 282–290. 24179783 10.1016/j.nicl.2013.01.012 PMC3777794

[22] Liu T, Surapaneni K, Lou M, Cheng L, Spincemaille P, et al (2012) Cerebral microbleeds: Burden assessment by using quantitative susceptibility mapping. Radiology 262: 269–278. 22056688 10.1148/radiol.11110251 PMC3244668

[23] Ritter F, Boskamp T, Homeyer A, Laue H, Schwier M, et al (2011) Medical image analysis: A visual approach. IEEE Pulse 2: 60–70. 22147070 10.1109/MPUL.2011.942929

